# The influence of season, hunting mode, and habitat specialization on riparian spiders as key predators in the aquatic-terrestrial linkage

**DOI:** 10.1038/s41598-023-50420-w

**Published:** 2023-12-22

**Authors:** Eric Bollinger, Jochen P. Zubrod, Dominic Englert, Nadin Graf, Oliver Weisner, Sebastian Kolb, Ralf B. Schäfer, Martin H. Entling, Ralf Schulz

**Affiliations:** 1 iES Landau, Institute for Environmental Sciences, RPTU Kaiserslautern-Landau, Fortstraße 7, D-76829 Landau, Germany; 2 Zubrod Environmental Data Science, Ostring 24a, D-76829 Landau, Germany; 3 Eußerthal Ecosystem Research Station, RPTU Kaiserslautern-Landau, Birkenthalstraße 13, D-76857 Eußerthal, Germany

**Keywords:** Ecosystem ecology, Riparian ecology, Stable isotope analysis

## Abstract

Freshwater ecosystems subsidize riparian zones with high-quality nutrients via the emergence of aquatic insects. Spiders are dominant consumers of these insect subsidies. However, little is known about the variation of aquatic insect consumption across spiders of different hunting modes, habitat specializations, seasons, and systems. To explore this, we assembled a large stable isotope dataset (n > 1000) of aquatic versus terrestrial sources and six spider species over four points in time adjacent to a lotic and a lentic system. The spiders represent three hunting modes each consisting of a wetland specialist and a habitat generalist. We expected that specialists would feed more on aquatic prey than their generalist counterparts. Mixing models showed that spiders’ diet consisted of 17–99% of aquatic sources, with no clear effect of habitat specialization. Averaged over the whole study period, web builders (WB) showed the highest proportions (78%) followed by ground hunters (GH, 42%) and vegetation hunters (VH, 31%). Consumption of aquatic prey was highest in June and August, which is most pronounced in GH and WBs, with the latter feeding almost entirely on aquatic sources during this period. Additionally, the elevated importance of high-quality lipids from aquatic origin during fall is indicated by elemental analyses pointing to an accumulation of lipids in October, which represent critical energy reserves during winter. Consequently, this study underlines the importance of aquatic prey irrespective of the habitat specialization of spiders. Furthermore, it suggests that energy flows vary substantially between spider hunting modes and seasons.

## Introduction

Riparian zones are interfaces that link aquatic and terrestrial systems, and because of their habitat complexity, are characterized by high productivity and biodiversity^1,2^. An important link between aquatic and terrestrial systems is the nutritional subsidy of terrestrial habitats via the emergence of aquatic insects, though the reciprocal energy flow, namely terrestrial-to-aquatic (e.g., in form of nutrients and organic matter^3^), is of comparable importance^4^. The significance of aquatic insects in the terrestrial food web owes itself not only to the quantity (i.e., multiple mg C m^−2^ yr^−1^ for average-sized lakes^5^), but also to the high nutritional quality that aquatic resources provide compared to terrestrial prey^4^. Aquatic systems are rich in energetically valuable substrates (e.g., periphyton^6^) and consequently emerging aquatic insects show high levels of essential highly polyunsaturated fatty acids (HUFA^7^).

Riparian spiders are an important node for the trophic linkage between aquatic and terrestrial ecosystems^8,9^ because they feed largely on aquatic insects^10,11^ but are also important predators of terrestrial arthropods^12^. Furthermore, spiders represent an important food source for consumers of higher trophic levels (e.g., birds and bats^13–15^) and compete with them for prey. The trophic niches of spiders, however, can be highly variable in space and time and are a function of their own and their prey’s traits as well as multiple environmental factors (e.g., temperature, riparian vegetation, and land use)^16–19^. More precisely, ground hunting spiders (GH) such as wolf spiders (Lycosidae) are known to feed strongly on terrestrial detritivore prey such as Collembola, with which they share a microhabitat^12,20,21^. By contrast, spiders hunting on vegetation (i.e., vegetation hunters, VH) such as Pisauridae likely feed more on herbivores^22^, while aerial web builders (WB) such as *Tetragnatha* sp. are expected to feed most strongly on actively flying aquatic insects, because a major fraction of this group has worse flying abilities than terrestrial flying insects^23^ and terrestrial prey includes entirely flightless groups (such as Collembola). In addition, the proportion of aquatic prey may be affected by the degree of specialization on riparian habitats. Firstly, wetland specialists could have evolved adaptations to better capture aquatic prey compared to habitat generalists. Second, the activity of wetland specialists is restricted to the surroundings of aquatic habitats, where aquatic prey is constantly available. By contrast, habitat generalists can move between upland and shoreline habitats, especially active ground hunters (e.g., *Pardosa* sp.). Thus, even individuals captured near water may have preyed on a more terrestrial diet in a different location.

In temperate regions, the aquatic subsidy is highly dynamic over time, peaking in summer^24,25^ due to the temperature-dependence of the development of subimaginal stages^26^. These patterns may shape the riparian food web since the diet of riparian spiders changes with relative food source availability^27^. Furthermore, spiders tend to hunt more actively at higher temperatures, and temperature can affect the web strength of web-building spiders^19,28^. In addition to spider traits and season, the proportion of aquatic prey may also differ between the surroundings of running and standing freshwater habitats, whereby the research is currently biased towards streams^5,29,30^. The few existing studies from lake ecosystems indicate that aquatic insects may have an even stronger and further reaching effect on terrestrial consumers than along stream ecosystems^31^.

With the aim to understand the variability in the utilization of aquatic subsidies by spiders, we compiled an extensive dataset (n > 1000) of stable isotope (SI) ratios of sources from aquatic and terrestrial origin as well as spider consumers. Samples were collected nearby lentic and lotic ecosystems (i.e., pond and stream, respectively) at four points in time (i.e., April, June, August, and October) to compare the dietary differences (i.e., the contribution of aquatic sources to spiders’ diet via Bayesian mixing models) between habitats and seasons. Since these seasonal patterns potentially differ with spiders’ hunting mode^11^, six spider species (Table 1) with three distinct hunting modes (i.e., web builder: WB, ground hunter: GH, and vegetation hunter: VH), were assessed. For each hunting mode, one wetland specialist and one habitat generalist were chosen^32^, respectively, to assess the potential effects of habitat specialization on the consumption of aquatic prey. It was hypothesized that the dietary proportions of aquatic prey are (1) higher in WB than in hunting spiders (i.e., GH and VH), (2) higher in wetland specialists than generalists, and (3) show seasonal changes (i.e., higher in seasons of high emergence). Furthermore, (4) potential differences between pond and stream were assessed.

**Table 1:** Included sources in mixing models based on spiders ‘ hunting mode.

Predator	Prey origin	Taxonomic level	Included taxa
Web builders (*Tetragnatha montana*^‡^, *Tetragnatha extensa*^†^)	Aquatic	Order	Coleoptera^Apr(P,S), Jun(P,S), Aug(P,S), Oct(P)^, Diptera^Apr(P,S), Jun(P,S), Aug(P,S), Oct(P,S)^, Ephemeroptera ^Apr(P,S), Jun(P,S), Aug(P,S), Oct(P,S)^, Hemiptera^Jun(P), Aug(P), Oct(P)^, Odonata^Aug(P), Oct(P,S)^, Plecoptera^Apr(P,S), Jun(S), Aug(P,S), Oct(S)^, Trichoptera^Apr(P,S), Jun(P,S), Aug(P,S), Oct(P,S)^
	Terrestrial	Order	Diptera^Apr(P,S), Jun(P,S), Aug(P,S), Oct(P,S)^
		Suborder	Auchenorrhyncha^Apr(P,S), Jun(P,S), Aug(P,S), Oct(P,S)^
		Family	Linyphiidae^Apr(P,S), Jun(P,S), Aug(P,S), Oct(P,S)^, Staphylinidae^Apr(P,S)^
Ground hunters (*Pardosa amentata*^‡^*, Pirata piraticus*^†^)	Aquatic	Order	Amphipoda^Apr(S), Jun(S), Aug(P,S), Oct(P,S)^, Anura^Apr(P), Jun(P)^, Caudata^Jun(P)^, Coleoptera^Apr(P,S), Jun(P,S), Aug(P,S), Oct(P)^, Diptera^Apr(P,S), Jun(P,S), Aug(P,S), Oct(P,S)^, Ephemeroptera^Apr(P,S), Jun(P,S), Aug(P,S), Oct(P,S)^, Hemiptera^Jun(P), Aug(P), Oct(P)^, Hydrachnidiae^Oct(P)^, Lumbriculida^Jun(P), Oct(P)^, Odonata^Aug(P), Oct(P,S)^, Oligochaeta^Apr(S)^, Onychura^Apr(P)^, Plecoptera^Apr(P,S), Jun(S), Aug(P,S), Oct(S)^, Trichoptera^Apr(P,S), Jun(P,S), Aug(P,S), Oct(P,S)^
	Terrestrial	Class	Collembola^Apr(P,S), Jun(P,S), Aug(P,S), Oct(P,S)^
		Order	Diptera^Apr(P,S), Jun(P,S), Aug(P,S), Oct(P,S)^
		Suborder	Auchenorrhyncha^Apr(P,S), Jun(P,S), Aug(P,S), Oct(P,S)^
		Family	Linyphiidae^Apr(P,S), Jun(P,S), Aug(P,S), Oct(P,S)^, Staphylinidae^Apr(P,S)^
Vegetation hunters (*Pisaura mirabilis*^‡^*, Dolomedes fimbriatus*^†^)	Aquatic	Order	Amphipoda^Apr(S), Jun(S), Aug(P,S), Oct(P,S)^, Anura^Apr(P), Jun(P)^, Caudata^Jun(P)^, Coleoptera^Apr(P,S), Jun(P,S), Aug(P,S), Oct(P)^, Diptera^Apr(P,S), Jun(P,S), Aug(P,S), Oct(P,S)^, Ephemeroptera^Apr(P,S), Jun(P,S), Aug(P,S), Oct(P,S)^, Hemiptera^Jun(P), Aug(P), Oct(P)^, Lumbriculida^Jun(P), Oct(P)^, Odonata^Aug(P), Oct(P,S)^, Oligochaeta^Apr(S)^, Plecoptera^Apr(P,S), Jun(S), Aug(P,S), Oct(S)^, Trichoptera^Apr(P,S), Jun(P,S), Aug(P,S), Oct(P,S)^
	Terrestrial	Class	Collembola^Apr(P,S), Jun(P,S), Aug(P,S), Oct(P,S)^
		Order	Diptera^Apr(P,S), Jun(P,S), Aug(P,S), Oct(P,S)^
		Suborder	Auchenorrhyncha^Apr(P,S), Jun(P,S), Aug(P,S), Oct(P,S)^
		Family	Linyphiidae^Apr(P,S), Jun(P,S), Aug(P,S), Oct(P,S)^, Staphylinidae^Apr(P,S)^

Superscripts indicate the presence of the taxa in the respective month (Apr = April, Jun = June, Aug = August, Oct = October) and system (P = Pond, S = Stream) as well as the specialization of the spiders (‡ = habitat generalist, † = wetland specialist).

## Material and methods

### Study site and sampling

**Figure 1:**
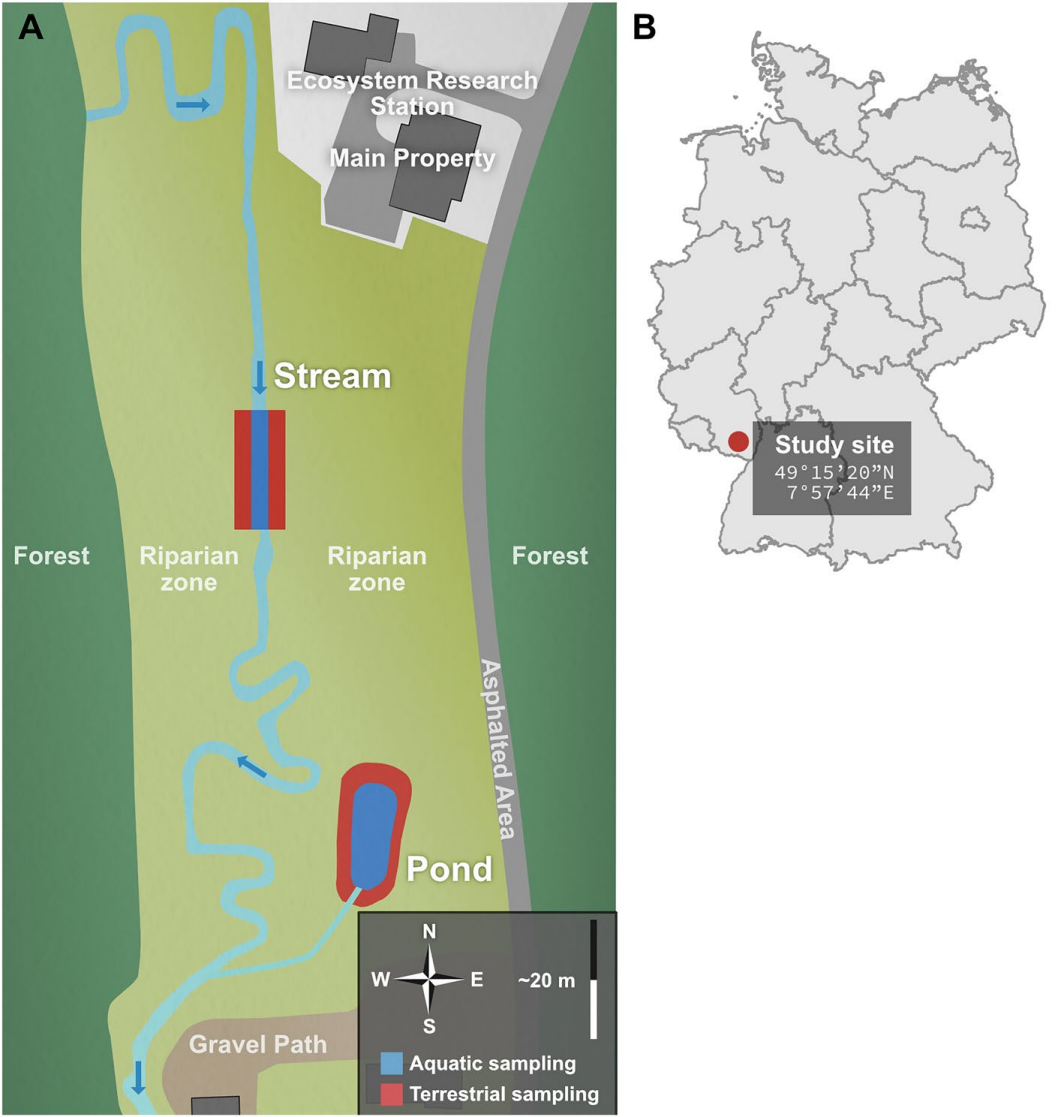
Areal perspective of the sampling site (**A**, not to scale) together with the location within Germany (**B**). Aquatic and terrestrial sampling sites are presented in dark blue and red, respectively. A compass and measure are included in the legend to provide cardinal direction and an approximate scale, respectively. Panel A was generated in Affinity Photo (1.10.6) and panel B used the R packages “ggplot2” (3.4.4) and “raster” (3.6.26).

Sampling took place at the Eußerthal Ecosystem Research Station (EERES; 49°15′20″N, 7°57′44E, Fig. 1) of the RPTU Kaiserslautern-Landau in Landau (see also^33^). Within EERES, a grassland area bordering a pond (lentic) and a small stream (lotic), respectively, were used. The pond had an area of ~ 17 m^2^ with flat, vegetated shores and a stable water level through its connection to a nearby source. The stream had an average width of 85 cm and an average depth of 30 cm with mostly sandy and occasional gravel sections, and flat shores. In April, June, August, and October of 2017, six spider species, as well as their potential prey, were sampled in both water bodies and their adjacent terrestrial areas (Table 1). Within each spider hunting mode, the wetland specialist has a narrower niche with the optimum in more moist habitats than the habitat generalist^32^. While also the habitat generalists (especially *Pardosa amentata* and *Tetragnatha montana*) prefer moist habitats, they can also be found away from water, while all wetland specialists are restricted to the surroundings of aquatic habitats^34^. Organisms were sampled all over the pond system and its surrounding riparian zone, whereas for the stream system a 20 m long stretch was sampled. Terrestrial systems were sampled up to 20 m away from the respective water body. The sampling of prey was not done quantitatively but aimed to include the complete set of potential prey items. Spiders were sampled with a suction sampler (Stihl SH 86) and sweep net or were collected by hand. After identification, they were frozen with liquid nitrogen. Potential terrestrial prey was sampled likewise. Preys were sorted into groups (see Table 1) directly in the field and afterward frozen with liquid nitrogen. Potential aquatic prey was sampled using emergence traps and kick sampling of benthic invertebrates. Seven emergence traps (basal area: 0.25 m^2^) per system were deployed for a week. The insects were caught with a bottle trap, filled with an aqueous solution of 1% (vol.) TWEEN® 80 and saturated with sodium chloride.

### Stable isotope and elemental analysis

Both spiders, and their potential prey were dried at 60 °C and ground to a fine powder. Afterward, approximately 0.5 mg of material was weighed (d = 0.0001 mg) into tin cups (5 mm × 9 mm; IVA, Meerbusch, Germany). If individual biomass was lower than 0.5 mg, several individuals of a taxon were combined into a pooled sample. The samples’ SI signatures and elemental content of N and C were determined in the Landau Stable Isotope Facility using a Delta V Advantage isotope ratio mass spectrometer coupled to a Flash HT Elemental Analyzer (Thermo Fisher Scientific, Bremen, Germany). SI signatures were expressed using the delta notation (δ; in per mil) relative to the respective international standards (atmospheric air for N and Vienna Pee Dee Belemnite for C). An internal reference material (i.e., casein) was measured in duplicate every ten samples with a precision (± 1 SD) of 0.05 and 0.03 for N and C, respectively.

### Calculations and statistics

Before modeling, several prey species were filtered out based on the spiders’ hunting mode (Table 1), which is considered good practice if done in an informed way^35^. For WBs only flying organisms were considered^36^, while for GHs and VHs also some Crustaceans and Anura were included, because *Pirata* and *Dolomedes* also hunt underwater^37,38^. Furthermore, since the dataset derived from kick samples is ~ 5 times richer than from emergence traps (i.e., because of the low sampling duration) and potential deviations due to isotopic fractionation during the metamorphosis^39,40^ are in an acceptable range, only the former was considered for mixing models. Since opisthosoma and prosoma show significant differences in their isotopic signatures (*p* < 0.001, hierarchical three-way ANOVA, Figure S1), only data from the former are considered for subsequent mixture modeling. This decision is based on the fact that opisthosomas tend to have, with approximately eight days, a shorter turnover rate (i.e., the time it takes a consumer to equilibrate with its sources’ isotope ratio) than other body parts of spiders^41^. Since sources and consumers were sampled on the same day opisthosomal SI signatures are thus more informative for the resource data of this study.

Subsequently, the proportions of aquatic sources to spider diets were separately estimated with Bayesian mixing models for each of the 48 combinations of time points (i.e., April, June, August, and October), system (i.e., pond and stream), and spider species (cf. Table 1) with a generalist prior using the R package “MixSIAR” (chainLength = 100,000, burn = 50,000, thin = 50, chains = 3, version 3.1.12, Stock et al.^42^). Model convergence was assessed via trace plots. To account for trophic enrichment, sources were corrected at 0.5 ± 0.19‰ for δ^13^C and 2.3 ± 0.24‰ for δ^15^N^43^. In due consideration of best mixing model practices^35^, terrestrial and aquatic sources were separately grouped into a maximum of six groups in total by k-means clustering based on within sum of squares. Cluster-specific weighted means and standard deviations were calculated based on the sample size of each source in the cluster. Models were only run if at least three (of a maximum of five) spider SI signatures were inside the resource polygon. No significance test was applicable. The models generate posterior probability distributions, and the discussion is based on their maximum a posteriori estimates (i.e., Bayesian equivalent of a mode, MAP) and 95%-highest-density credible intervals. The R code^44^ and the raw data can be found at https://doi.org/10.7910/DVN/NSVHCN.

### Ethical approval

All experimental protocols were conducted under permit of the department 42 of the Struktur- und Genehmigungsdirektion Süd (raft spider, *Dolomedes fimbriatus*, n = 58, 42/553-254/281-17 and European common toad, *Bufo bufo*, n = 10, 42/553-254/ 354-17) and were carried out in accordance with relevant guidelines and regulations (i.e., ARRIVE). The respective ARRIVE guideline statement is included in section 2 of the supplemental information.

## Results

### Ground hunters

**Figure 2:**
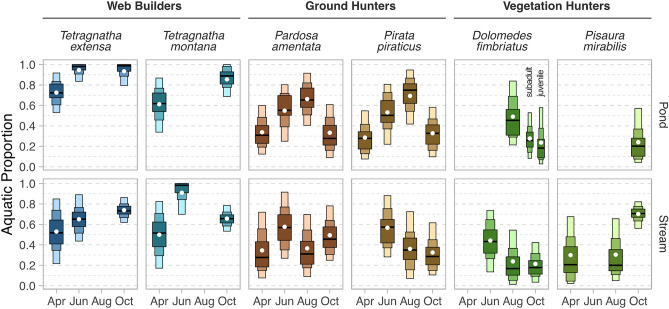
50% (light), 75% (medium), and 95% (dark) highest density intervals of the posterior distribution of aquatic proportion to spiders’ diet. Black lines show the maximum a posteriori probability and white dots the median of the posterior distribution. The seasonal data is shown for ground hunters, web builders, and vegetation hunters in the riparian system adjacent to the pond and the stream. For each feeding type, one species is considered a wetland specialist (i.e., *Tetragnatha extensa*, *Pirata piraticus*, and *Dolomedes fimbriatus*) while the other is considered a habitat generalist (i.e., *Tetragnatha montana, Pardosa amentata*, and *Pisaura mirabilis).* If the model could not be fitted, no crossbar is shown.

**Figure 3:**
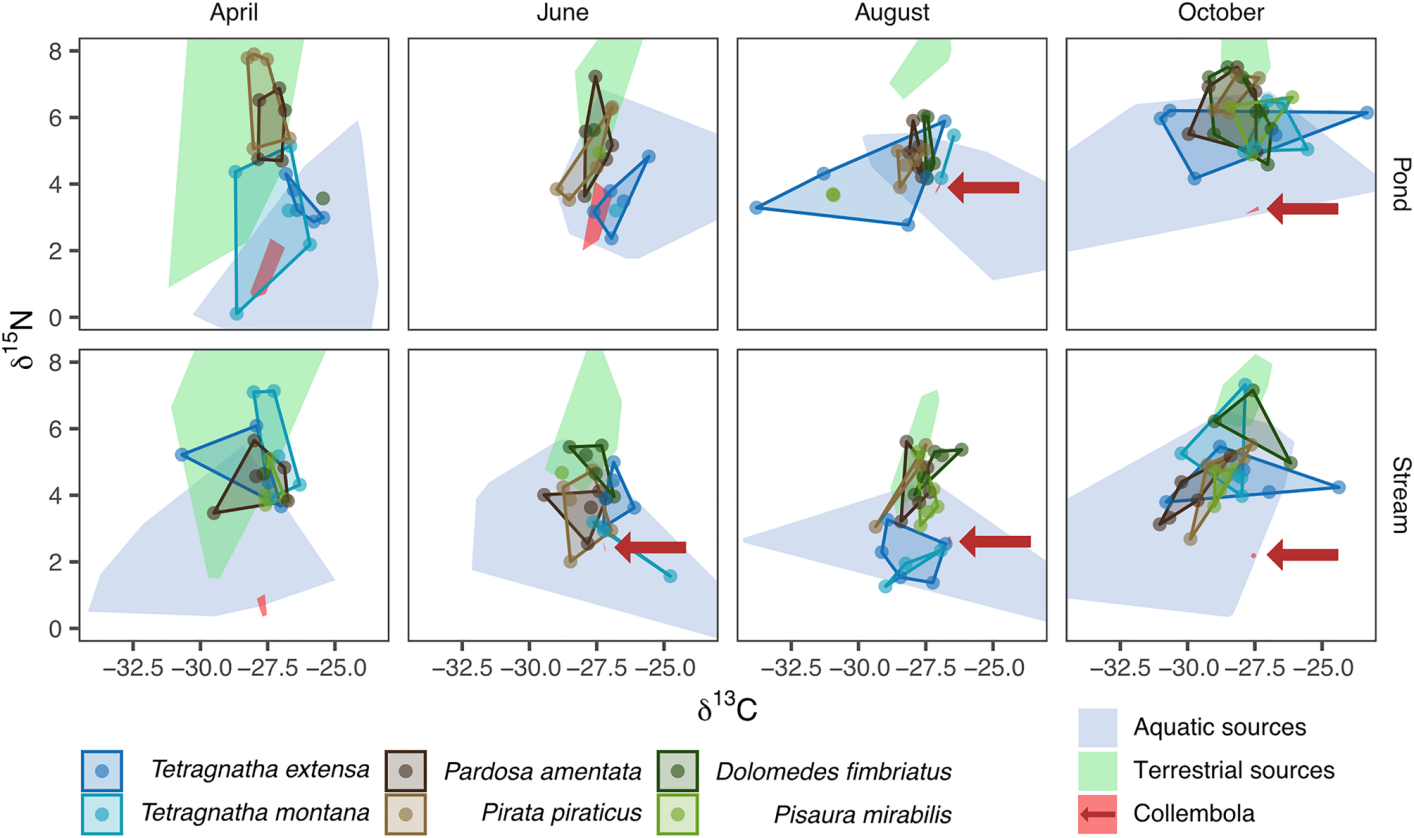
Stable isotope signatures of spiders’ opisthosomata (points with solid hull) and prey corrected for trophic enrichment (areas). Data is separated for each month (columns) and system (rows). To improve readability, Collembola are indicated by a red arrow if the data is hardly visible.

GHs represent the richest dataset of this study with only one of 16 possible mixing models not being fitted due to insufficient sampling of spiders. In the riparian habitat adjacent to the pond, MAPs of assimilated aquatic prey by *Pardosa amentata* and *Pirata piraticus* were 31% (12–60%) and 28% (8–55%) in April, respectively, which is in a comparable range to the 28% (8–72%) estimated for the riparian habitat adjacent to the stream (Fig. 2). The aquatic contribution to their diet increased to 50–55% (22–81%) in June and 65–75% (41–95%) in August next to the pond, however, with notably lower percentages in the habitat adjacent to the stream (31–34%; 7–73%). In October, the proportion of aquatic diet declined to 28–46% (9–78%). Within each system, however, both species occupy a comparable dietary niche based on SI signatures (Fig. 3). C:N ratios changed significantly throughout the year (*p* < 0.001; F = 90.2; df = 3; ANOVA) and were notably higher in October, which was the case for all species assessed.

### Web builders

WBs represent the second richest dataset of this study with four mixing models not being fitted due to insufficient sampling and two because consumer SI signatures were outside the resource polygon. In the habitat adjacent to the pond, the proportion of aquatic sources to the WBs’ diet were 72% (53–92%) and 62% (34–87%) for *Tetragnatha extensa* and *Tetragnatha montana*, respectively, in April (Fig. 2). The values were in a comparable range adjacent to the stream with 52% (22–85%) and 52% (17–82%) for *T. extensa* and *T. montana*, respectively (Fig. 2). *T. extensa* and *T. montana* almost exclusively fed on aquatic prey in June (i.e., MAPs between 65 and 98%; 44–100%). No models were fit for the data from August because too many spider isotope signals were outside the resource polygon. In August, driven by the mayfly species *Rhithrogena sp.,* the resource polygon covered less negative δ^13^C values compared to October. This taxon was absent from kick samples in August but showed a distinct isotope signal (δ^13^C < − 39‰) in October and was also found in April and June. In October, aquatic dietary proportions of WBs adjacent to the pond remained high (MAP > 89%; 69–100%) compared to WBs next to the stream (MAP < 73%, 53–86%). Within each system, SI signatures of both WB species were associated with highly variable niche sizes (i.e., area covered by SI signatures) between time points (Fig. 3).

### Vegetation hunters

VHs represent the smallest dataset of this study with seven of 16 mixing models not being fitted due to insufficient sampling of spiders. Aquatic proportions to their diet showed comparatively little fluctuations at a lower level (mean MAP = 31%; 12–65%) compared to what was observed for GHs and WBs (Fig. 2). In cases where sufficient data were available (i.e., August stream, October pond, October stream), SI signatures were comparable between species with slightly higher δ^15^N values (i.e., a proxy for trophic position) for *Dolomedes fimbriatus* than *Pisaura mirabilis* (Fig. 2).

## Discussion

### Comparison of hunting modes

Mixing models suggest that every spider species utilized an ecologically significant amount of prey from aquatic sources, but with major differences between hunting modes. As hypothesized, WBs showed the highest aquatic proportion in their diet averaged over the whole study duration followed by GHs and VHs. The diet of WBs occurring adjacent to surface waters is known to depend strongly on aquatic insects since their webs almost exclusively catch flying insects^36^. The seasonal increment in aquatic subsidy was highest in WBs and GHs. For WBs this is probably a result of a higher prevalence of aquatic insects ending up in their webs leading to an almost exclusively aquatic diet during the summer season. On the other hand, since GHs actively hunt their prey, both increased aquatic prey density (i.e., “chance-hypothesis”) and selective feeding on aquatic insects (i.e., “choice-hypothesis”) could explain the observed increment. Given that spiders’ ability to feed selectively tends to increase with increasing prey availability^45,46^ (but see^47^), the two hypotheses are not mutually exclusive. However, for GHs and VHs, inference from the data is complicated by the clustering of Collembola with aquatic sources (Fig. 3). Species of this class mainly assimilate matter from fungi, algae, and detritus^48–50^, which are low in ^15^N. Thus, Collembola were consistently lower in their δ^15^N signatures than other terrestrial prey groups. Assuming that Collembola make up a large fraction of GHs’/VHs’ diet (as indicated by a meta-analysis^11^) this could inflate the estimated dietary contribution from aquatic sources, which complicates the comparison between the assessed spider species. Since the clustering of sources can also increase the uncertainty and bias of mixing models^51,52^, comparisons of habitat specialization and seasonality within each hunting mode might also be affected by the presence of Collembola.

### Comparison of habitat specialization

Against our hypothesis, mixing models do not support relevant species-specific differences in the proportion of aquatic prey within each hunting mode. This result indicates that for the case assessed (i.e., aquatic vs. terrestrial) prey availability, which is influenced by the habitat structure^53^, is likely more important to a species’ diet than habitat specialization. This fits with the fact that most spiders are prey generalists, including the species studied here^10^. Selective feeding of habitat specialists could occur if they employ a different searching behavior or occupy different microhabitats as an adaptation to the aquatic prey that is common in their habitats, but we did not find any indication for this. However, the lack of detection of such differences could also be due to the underdetermination (i.e., number of tracers < number of sources + 1) of SI mixing models increasing the uncertainty of dietary contributions, which is an accurate and desirable feature^35^. Therefore, more subtle differences in prey selection could still be assessed through further tracers like fatty acids^42,54,55^, molecular analysis of the spiders’ gut content via species-specific primers^56^ (if resources are known) or sequencing of spider guts^57^ (if resources are unknown). Nevertheless, based on the dietary contribution of aquatic and terrestrial sources (Fig. 2) and SI niches (Fig. 3), the data of this study provided evidence against dietary differences with the degree of specialization.

In this context, it is important to note that from April to August, all *D. fimbriatus* analyzed were young individuals, which are vegetation-dwelling like the generalist *Pisaura mirabilis*. In the study region, *D. fimbriatus* have a 2-year development, in which subadult and adult individuals adopt a more ground-dwelling lifestyle and frequently hunt for large prey (including tadpoles and fish) on or even below the water surface, earning them the common name “fishing spider”^38,58^. Thus, the similar dietary niche between *Dolomedes* and *Pisaura* that we found may be restricted to the life stages that we examined. Only in October, we sampled a mixture of young and subadult *Dolomedes* individuals. However, neither age group of *Dolomedes* had elevated aquatic prey signals compared to the earlier months or to *Pisaura* (Fig. 2). Possibly, a higher reliance of *Dolomedes* on aquatic prey only develops during the reproductive period (i.e., after winter).

### Seasonality in aquatic prey utilization

As hypothesized, for all species, the utilization of aquatic prey was higher during summer (i.e., June and August) with the highest increments for GHs and WBs. This increase is most likely triggered by the onset of the emergence of aquatic insects or a reduction in terrestrial prey availability. Given that aquatic sources have on average at least 2.19‰ lower δ^15^N values than terrestrial prey (Fig. 3), δ^15^N values of spider consumers (i.e., an indicator of trophic position^59^) predominantly show a reciprocal pattern to the seasonal changes in the contribution of aquatic sources (Fig. 3). This pattern suggests that ignoring cross-ecosystem subsidies could bias inferences on trophic positions in the subsidized habitat^60^. Hence, the use of two-baseline models^61^ is essential in the aquatic-terrestrial meta-ecosystem.

**Figure 4:**
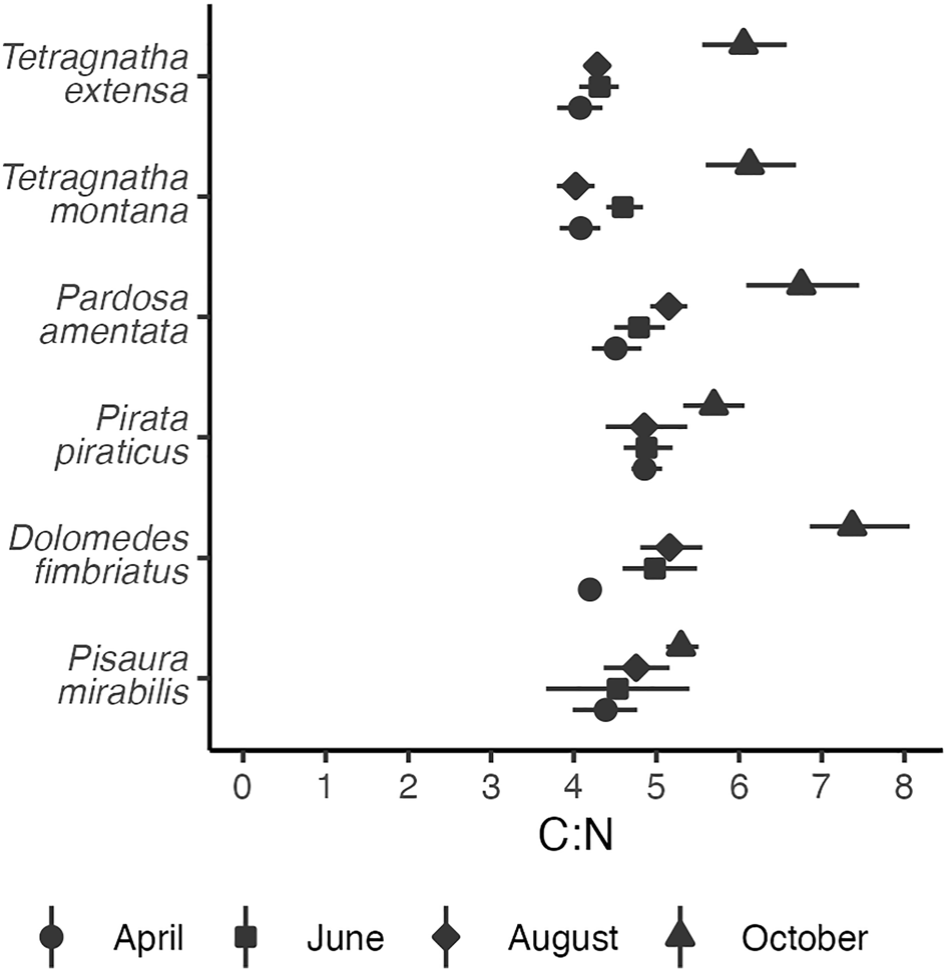
Bootstrapped means with 95% confidence intervals of C:N ratios of spiders’ opisthosomata in April (circles), June (squares), August (diamonds), and October (triangles).

Furthermore, C:N ratios of all spider species were notably higher in October (Fig. 4). Although this could also be explained by the fall of leaves that typically show high C:N ratios (data not shown) it is more likely an indicator of a higher proportion of lipids^62^. Likewise, it could be interpreted as an accumulation of lipids^63^ that serve as an energy reserve for the winter season^64^. This implies that the assessed spider species are able to shift from a somatic growth strategy in summer to an energy storage accumulation strategy in the fall^63^. Given that the fatty acid profile of a consumer is a result of its diet^65^ and determines diapause characteristics^66,67^, the dietary quality in this season might be of particular importance. For example, cryoprotectants such as glycerin are central to the cold-tolerance^64^ and are allocated via lipolysis^68^. Consequently, the survival of spiders and thus their ecological role in the subsequent year could be to a large degree determined by aquatic subsidies and their alterations, especially during fall, which underlines the fallacy of assessing subsidies quantitatively without considering dynamics^69–71^.

### Comparison of lotic and lentic systems

Following the conceptual model of Gratton and Vander Zanden^5^, for both lakes and streams, aquatic emergence fluxes scale linearly with ecosystem size. Ignoring potential differences in the productivity of these systems, this model would suggest a higher flux of emergence from the pond for the present study. However, water velocity and emergence are typically positively correlated^72^, which can be mechanistically explained by increasing suspended particles and many emerging insects being collector-filterers. Thus, both higher, or lower use of aquatic subsidies are feasible next to lentic and lotic ecosystems (compared to the respective other). The lack of a clear pattern in our data (Fig. 2) suggests that differences (if present) are indeed minor. However, due to the proximity of the systems to one another in our study, we cannot exclude that movement of prey sources between both systems has blurred possible differences.

## Conclusion

All spider species assessed were subsidized by aquatic emergence to an ecologically relevant degree that increases in seasons of high emergence. The use of aquatic prey was much more determined by hunting mode than by habitat specialization, being highest for WBs followed by GHs. The lack of an effect of habitat specialization is in accordance with the generalist feeding and low prey selectivity of most spider species. C:N ratios suggest the accumulation of lipids in fall. This season might be of particular importance for the nutrition of spiders, which strongly underlines the need to consider the temporal dynamics when evaluating the relevance of aquatic subsidy for the terrestrial food web together with its magnitude.

### Supplementary Information

Supplementary Information.

## Data Availability

Raw data is available at https://doi.org/10.7910/DVN/NSVHCN.

## Code availability

Code is available at https://doi.org/10.7910/DVN/NSVHCN.
