# Supplementary material for: The influence of season, hunting mode, and habitat specialization on riparian spiders as key predators in the aquatic-terrestrial linkage
Source: Sci Rep. 2023 Dec 22;13:22950. doi: 10.1038/s41598-023-50420-w (PMC10746743; doi:10.1038/s41598-023-50420-w)
Supplement: Supplementary file 1 — Supplementary Information. [file 41598_2023_50420_MOESM1_ESM.docx]

**Supplemental Information:**

**Riparian spiders as key predators in the aquatic-terrestrial linkage: role of season, hunting mode, and habitat specialization**

Eric Bollinger^1,*^, Jochen P. Zubrod^1,2^, Dominic Englert^1^, Nadin Graf^1^, Oliver Weisner^1^, Sebastian Kolb^1^, Ralf B. Schäfer^1^, Martin H. Entling^1^, Ralf Schulz^1,3^

^1^iES Landau, Institute for Environmental Sciences, RPTU Kaiserslautern-Landau, Fortstraße 7, D-76829 Landau, Germany

^2^Zubrod Environmental Data Science, Ostring 24a, D-76829 Landau, Germany

^3^Eußerthal Ecosystem Research Station, RPTU Kaiserslautern-Landau, Birkenthalstraße 13, D-76857 Eußerthal, Germany

**SI.1 Figures**

**Figure S1:** Bootstrapped means with 95% confidence intervals of stable isotope signatures of spiders’ opisthosomata (blue) and prosomata (orange) in April (circles), June (squares), August (diamonds), and October (triangles).

**Figure S2:** Seasonal temperature profile of the stream. Measurements are plotted as semi-transparent circles and temperature is smoothed via a generalized additive model (blue line).


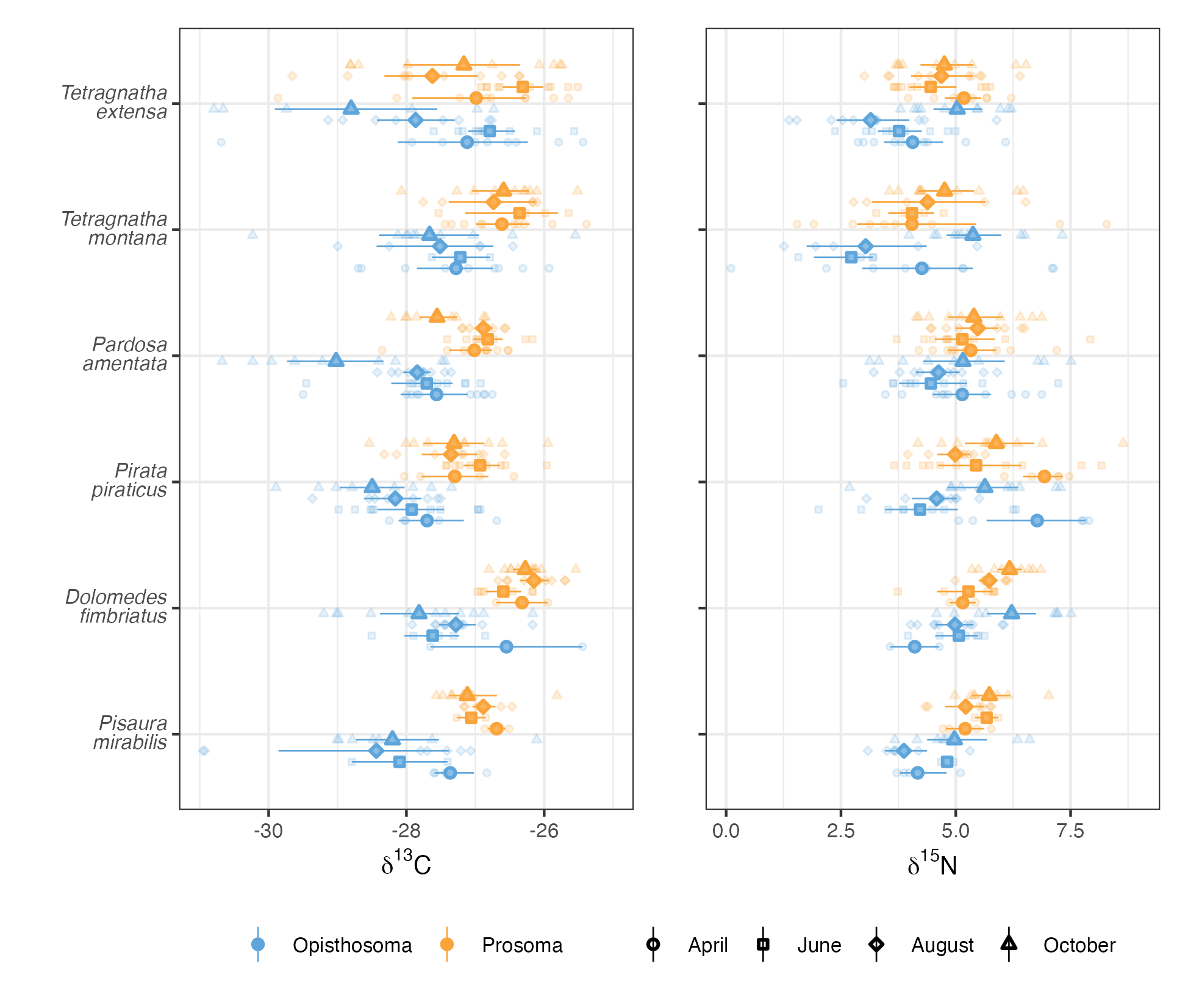


**Figure S1**


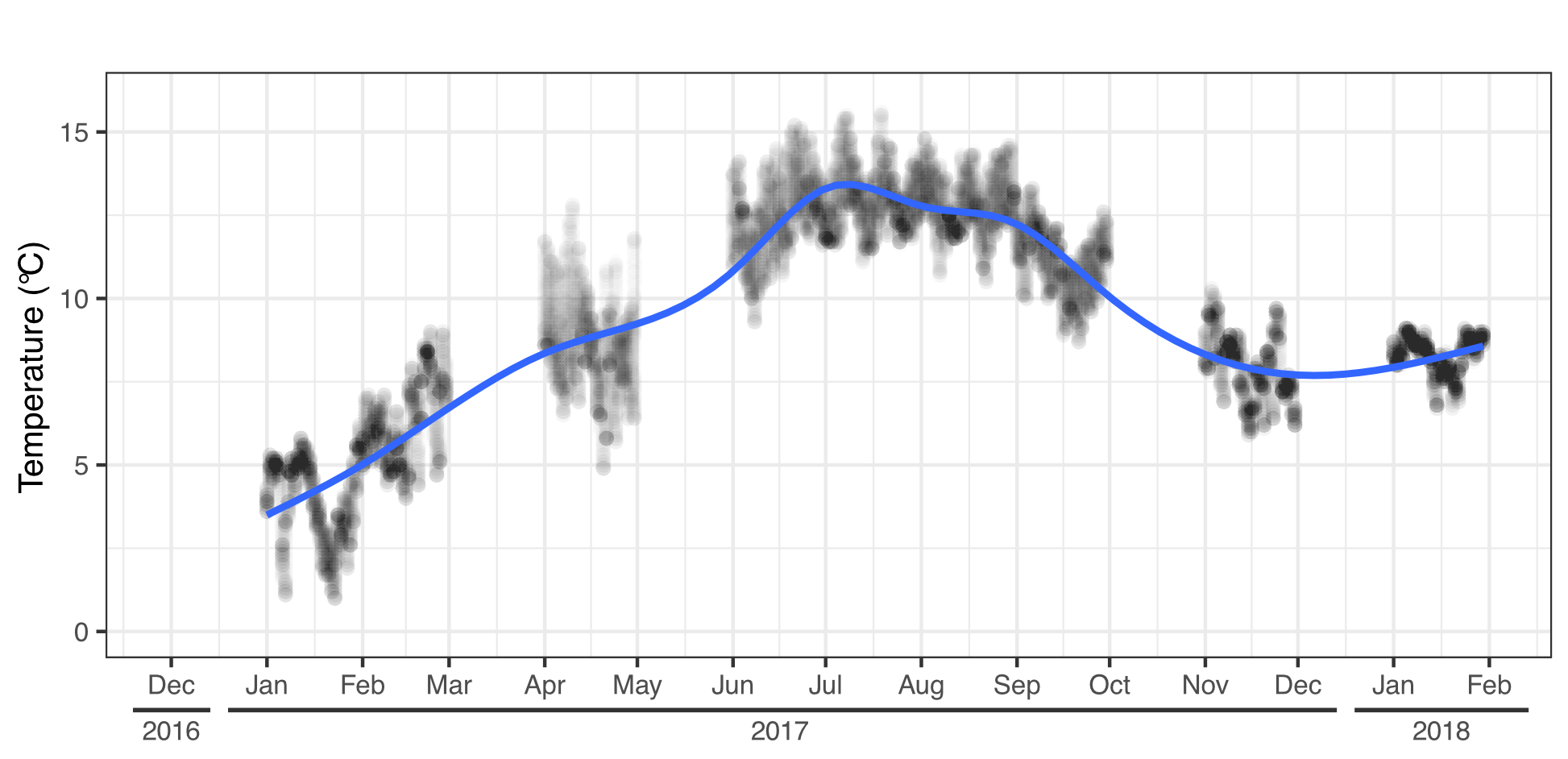


**Figure S2**

**SI.2 ARRIVE guideline statement**

1. Study design:
   1. Experimental design:
      *Since this is an observatory field study, no treatments have been applied, Therefore, also a control is missing.*
   2. Experimental unit:
      *The experiment was conducted in a natural stream and pond and the adjacent riparian zone. Whole organisms were sampled from the natural population.*
2. Sample size:
   1. Number and distribution of samples:
      *In total, 58 samples of the raft spider,* Dolomedes fimbriatus *and 10 samples of European common toad,* Bufo bufo *were used.* B. bufo *were only sampled from the pond in April (n=5) and June (n=5). D. fimbriatus distribute the following:*

| **System** | **Month** | **n** |
| --- | --- | --- |
| Pond | April | 2 |
|  | June | 2 |
|  | August | 10 |
|  | October | 16 |
| Stream | April | 2 |
|  | June | 10 |
|  | August | 10 |
|  | October | 6 |
|  |  | Σ 58 |

- 1. Sample size decision:
     *We aimed for 5 replicates for resources of interest and 10 for consumers. In October, we sampled* D. fimbriatus *of different sizes to account for differences in their diet, which resulted in 16 replicates. This replication was based on the applied mixing models for diet reconstruction.*

1. Inclusion and exclusion criteria:
   1. Inclusion:
      *None.*
   2. Exclusion:
      *None.*
2. Randomization:
   1. Randomization strategy:
      *No randamization was necessary since natural populations were observed.*
   2. Confounder reduction strategy:
      *No confounders were considered.*
3. Blinding:
   *Blinding is not applicable for a field study. Further analysis (i.e., stable isotope analysis) are also not prone to error because of this.*
4. Outcome measures:
   1. Definition of outcome measures:
      *The following endpoints were assessed: δ^15^N, δ^13^C, %N and %C. All these endpoints were measured from the same sample.*
   2. Hypothesis testing outcome measure:
      *Bayesian mixing models are not used for frequentist hypothesis testing.*
5. Statistical methods:
   1. Methods and Software:
      *Mixing models were done using the “R” package “MixSIAR” (version 3.1.12).*
   2. Model assumptions:
      *Mixing models assume that provided data are the entire ecologically relevant food sources, trophic enrichment factors are applicable to the model consumer and that the physical principle of mass balance is followed. The former two were accomplished by the sampling effort and literature analysis, while the latter is a fundamentally true property of nature.*
6. Experimental animals:
   1. Details:
      *Juvenile* B. bufo *and subadult/adult* D. fimbriatus *were sampled. Their sex was not determined. Weights of some* D. fimbriatus *were measured and ranged from 0.37 to 62.14 mg (median: 2.5 mg).*
   2. Provenance:
      Organisms were obtained from the natural pond and stream and the adjacent riparian zone.
7. Experimental procedures:
   1. What was done?
      *Organisms were kick sampled and frozen in liquid nitrogen.*
   2. When and how often?
      *This is elaborated in Section 2.a*
   3. Where?
      *Organisms were obtained from the natural pond and stream and the adjacent riparian zone.*
   4. Why? *Organisms need to be killed before drying and subsequent stable isotope measurements. Otherwise, the research goal would not have been possible*
8. Results:
   1. Summary:

|  | **Endpoint** | | | |
| --- | --- | --- | --- | --- |
| **Organism** | **δ^15^N (‰)** | **N (%)** | **δ^13^C (‰)** | **C (%)** |
| *D. fimbriatus* | 5.59±0.87 | 9.96±2.35 | -26.9±0.88 | 44.98±7.25 |
| *B. bufo* | 1.79±0.33 | 5.65±2.11 | -27.7±1.62 | 26.72±9.22 |

- 1. Effect sizes:
     *Not applicable.*
